# Different Effects of Structured Education on Glycemic Control and Psychological Outcomes in Adolescent and Adult Patients with Type 1 Diabetes: A Systematic Review and Meta-Analysis

**DOI:** 10.1155/2020/9796019

**Published:** 2020-02-26

**Authors:** Fang Liu, Yuzhu Guan, Xia Li, Yuting Xie, Jing He, Zhi-Guang Zhou, Lezhi Li

**Affiliations:** ^1^Xiangya School of Nursing, Central South University, Changsha, Hunan Province, China; ^2^Department of Metabolism and Endorinology, The Second Xiangya Hospital, Central South University, Changsha, Hunan Province, China; ^3^Medical Psychological Center, The Second Xiangya Hospital, Central South University, Changsha, Hunan Province, China; ^4^National Clinical Research Center for Metabolic Disease, Changsha, China; ^5^Clinic Nursing Teaching and Research Section, The Second Xiangya Hospital, Central South University, Changsha, Hunan Province, China

## Abstract

**Aim:**

This systematic review aimed at investigating the effectiveness of structured education (SE) in improving glycemic control and psychological outcomes in adolescent and adult patients with type 1 diabetes.

**Methods:**

Electronic databases (EMBASE, Medline, PubMed, and the Cochrane Library) and the reference lists of included studies were searched from the beginning of the database through April 2019. Randomized controlled trials comparing SE with a control condition and reporting a change in glycosylated hemoglobin (HbA1c) level were included. The primary outcome was glycemic control measured by HbA1c. Secondary outcomes were diabetes-related distress, well-being, depression, and quality of life.

**Results:**

Eighteen studies representing 2759 patients were included. Twelve studies targeted adolescents and six targeted adults. Adolescent patients who were randomized to the intervention group did not show significant improvement of HbA1c in the short (SMD = −0.04; 95% CI: −0.14 to 0.06; *P*=0.41), medium (SMD = −0.03; 95% CI: −0.13 to 0.07; *P*=0.41), medium (SMD = −0.03; 95% CI: −0.13 to 0.07; *P*=0.41), medium (SMD = −0.03; 95% CI: −0.13 to 0.07; *P*=0.41), medium (SMD = −0.03; 95% CI: −0.13 to 0.07; *P*=0.41), medium (SMD = −0.03; 95% CI: −0.13 to 0.07;

**Conclusions:**

Development of more efficient SE programs according to the patients' personal characteristics is needed.

## 1. Introduction

Type 1 diabetes mellitus is a chronic condition characterized by an absolute insulin deficiency and a lifelong dependency on exogenous insulin [1]. According to the International Diabetes Federation (IDF), there were approximately 425 million adults suffering from diabetes mellitus (DM) globally in 2017, of which approximately 5% to 10% had type 1 diabetes [2]; the incidence of type 1 diabetes in adolescents is on the rise and is 6.5% in China [3, 4]. While daily administration of insulin and lifestyle modifications [5–7] have dramatically changed the disease prognosis in recent decades, the dosing adjustments of intensive insulin therapy and reductions in glycosylated hemoglobin (HbA1c) in order to minimize diabetes-related complications are still challenging in these patients with type 1 diabetes [8, 9].

Diabetes self-management education is a crucial element in the treatment of diabetes. Notably, it can improve patients' self-management skills and prevent long-term complications [10, 11]. Among a number of diabetes education programs, structured education (SE) was considered to be a high-quality education program for patients with type 1 diabetes [12, 13].

Since an adapted SE program (Dose Adjustment For Normal Eating, DAFNE) for patients with type 1 diabetes in the UK was evaluated using a randomized controlled trial and reported a significant improvement in both glycemic control and quality of life [14], SE has been adopted by the UK National Institute for Clinical Excellence (NICE) and the UK Department of Health [15, 16]. NICE defines the SE as “a planned and graded program that is comprehensive in scope, flexible in content, responsive to an individual's clinical and psychological needs, and adaptable to his or her educational and cultural background” and recommends SE for all people with diabetes, stating that it should meet five quality criteria: (1) have a structured written curriculum, (2) have a patient-centered philosophy, (3) is led by trained educators, (4) meets quality assurance standards, and (5) is audited. In many countries, SE has become standard practice for patients with type 1 diabetes [17, 18].

Although SE is widely accepted as an effective tool for patients with type 1 diabetes to manage their conditions, the conclusions of various studies were inconsistent. Some studies reported that structured type 1 diabetes education can improve glycemic control and quality of life (QOL), reducing the incidence of some severe diabetes-related complications [19–21] and had a cost-effective benefit and long-term positive effect in adults [22, 23]. However, some studies reported that glycemic control did not improve after the implementation of SE [24, 25], especially for adolescents [26–28]. In some studies, there was a trend for HbA1c to return to baseline levels in the long term [29, 30].

Until recently, no meta-analysis of randomized controlled trials (RCTs) examining the effectiveness of SE for patients with type 1 diabetes has been published. There is also no meta-analysis examining the long-term effect of SE on patients with type 1 diabetes. To overcome these limitations, we performed a comprehensive systematic review and meta-analysis of RCTs to examine the effectiveness of SE on glycemic control in the short, medium, and longer term in patients with type 1 diabetes. We also explored the effectiveness of SE on various psychological outcomes. The results of this systematic review and meta-analysis may be incorporated into the available evidence and guide future research in this field.

## 2. Methods

This meta-analysis was completed in compliance with the Preferred Reporting Items for Systematic Reviews and Meta-Analyses (PRISMA) statement [31].

### 2.1. Search Strategy

We searched the PubMed, Medline, EMBASE, and the Cochrane Library databases from the beginning of the database through April 2019, with no geographical area restriction. The reference lists of included studies were also searched by hand for additional studies. To identify RCTs, we adopted a highly sensitive literature search strategy, using a combination of free-text words and Medical Subject Headings (MeSH) terms. The search strategies for PubMed were as follows: (“diabetes mellitus type 1” [MeSH Terms] OR type 1 diabetes OR insulin-dependent diabetes OR T1DM) AND (structured education OR “education” [MeSH Terms] OR structured educational program OR structured health education OR structured diabetes education) AND (randomized controlled trial OR controlled clinical trial OR random allocation OR random OR randomly OR randomized OR single-blind OR double-blind).

Two reviewers independently examined titles and abstracts of articles identified from the search. Only clearly irrelevant studies were discarded. The full text of studies that seemed to be potentially eligible was retrieved. The same two reviewers independently read the full text of studies and selected studies that met the inclusion criteria. In cases of disagreement regarding inclusion, consensus was reached based on discussion by the authors.

### 2.2. Eligibility Criteria

Studies were included based on the following inclusion criteria: (1) RCTs comparing SE with a control condition (usual care, waiting list, or attention control); (2) studies meeting the key criteria of SE defined by NICE; (3) the study population was diagnosed with type 1 diabetes; (4) HbA1c was measured as the primary outcome or as part of multiple outcomes; and (5) studies published in English. The exclusion criteria were (1) studies combining type 1 and type 2 diabetes and (2) trials for which full-text reports were not available. There was no limitation on the year of publication, duration of intervention, or the length of follow-up. For studies that reported the results in multiple follow-up times, we included the longest follow-up trial. When studies provided insufficient data on HbA1c, we contacted the author by email. If no response was received, the study was excluded.

### 2.3. Assessment of Risk of Bias

The quality of the included studies was assessed independently by two reviewers using the criteria of the Cochrane Collaboration risk of bias assessment tool [32], including judgment of sequence generation, allocation concealment, completeness of outcome data, selective outcome reporting, and other sources of bias. Since blinding of participants and personnel was not feasible for trials of SE, we removed this item from the assessment and divided the blinding of outcome into objective outcome (HbA1c) and subjective outcome (psychological outcomes). Disagreements were resolved by discussion.

### 2.4. Data Extraction

Two reviewers independently extracted the following information from included studies: general study information, baseline characteristics of participants, characteristics of interventions, time points of outcome assessments, and baseline and follow-up outcome data. Missing information was requested from the corresponding authors. If the trials had more than two groups, we extracted only the information and data of interest reported in the original articles. Disagreements were resolved by consensus.

The primary outcome was glycemic control measured by glycated hemoglobin (HbA1c). Secondary outcomes were diabetes self-efficacy, diabetes-related distress, well-being, depression, and QOL.

### 2.5. Data Synthesis and Analysis

Results for the mean change in HbA1c, scores of diabetes-related distress, well-being, and depression measured by scales before and after the intervention were pooled in a meta-analysis using Review Manager 5.3, and results for QOL were analyzed through narrative synthesis due to insufficient data and the various scales used. Not all measurements were reported in the same form, so we calculated some of the results. A standardized mean difference with 95% confidence intervals for continuous data was used to estimate the effect size. We quantified heterogeneity between studies by using the chi-square test and Higgins' *I*^2^ test. We considered heterogeneity to be low and a fixed-effects model was used if *P* > 0.1 and *I*^2^<50%, and a random-effects model was used if *P* < 0.1 and *I*^2^ was between 50% and 75% [32]. Subgroup analyses were planned for age (adults or youth), follow-up time, and baseline HbA1c (%) to evaluate the treatment effects and explain any heterogeneity.

## 3. Results

### 3.1. Study Selection

**Figure 1:**
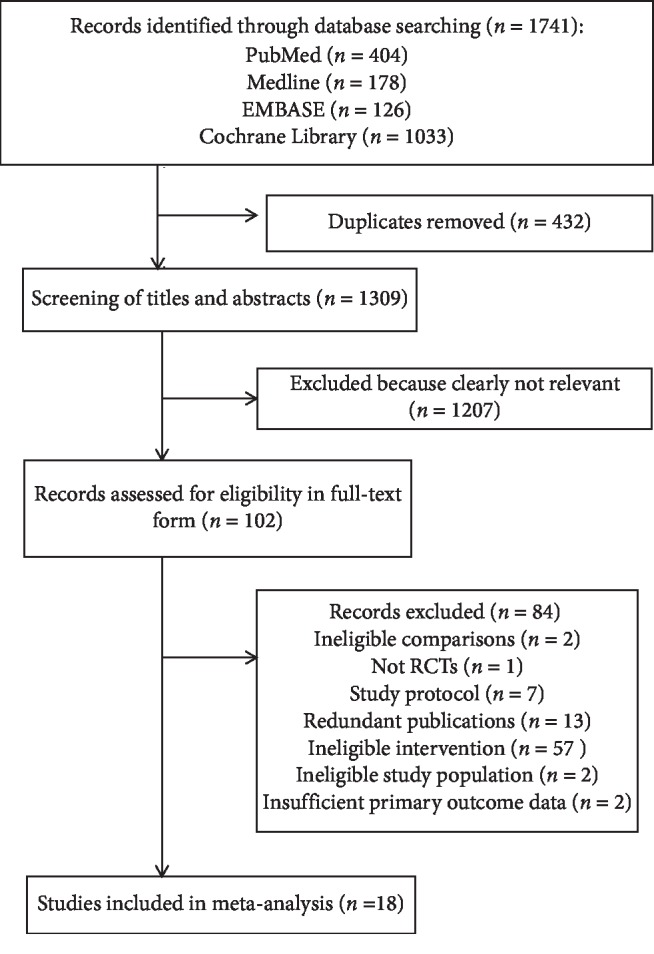
Flow diagram of study selection.

The electronic search and additional hand-searching retrieved 1741 publications, and after removal of duplicates, 1309 records remained. Of these, 102 were identified as potentially eligible studies on the basis of title and abstract. Full texts of these records were reviewed for further examination, leading to a total of 18 RCTs included in this meta-analysis. The detailed study selection process is documented in Figure 1.

### 3.2. Characteristics of the Included Studies

Eighteen studies fulfilled the inclusion criteria [14, 25–28, 33–45], and the characteristics of the included studies are presented in Table 1. All included studies were published between 2000 and 2016. Sixteen of the included studies used a parallel design [14, 25, 28, 33–45], and two employed a cluster randomized design [26, 27]. There were twelve studies targeting adolescent patients [26–28, 34–36, 38, 40–42, 44, 45] and six including adult patients [14, 25, 33, 37, 39, 43]. The sample sizes of the included studies ranged from 53 to 396. The mean (SD) age of the 2759 study participants ranged from 9.9 (1.4) to 46.3 (13.8) years, and the mean baseline HbA1c was between 7.0 (1.3) and 10.0 (1.5). The interventions across studies varied in format, and most studies used face-to-face group education sessions. The length of the intervention varied between five days and fifteen months. With regard to control groups, the majority of included RCTs used usual care [26–28, 33, 35–37, 39, 41–45], two studies used wait-list control [14, 40], and three studies used attention control [25, 34, 38]. All studies except one provided detailed information on the control group [40]. In terms of outcomes, all studies reported HbA1c as an outcome measurement, four measured diabetes self-efficacy [27, 33, 34, 38], five measured diabetes-related distress [33–35, 37, 39], three measured depression [33, 37, 39], three measured well-being [14, 35, 37], and eleven measured quality of life [14, 25–27, 34, 35, 38–40, 42, 43]. The follow-up time ranged from immediately after the intervention to 24 months after the intervention.

### 3.3. Risk of Bias Assessment Results

**Figure 2:**
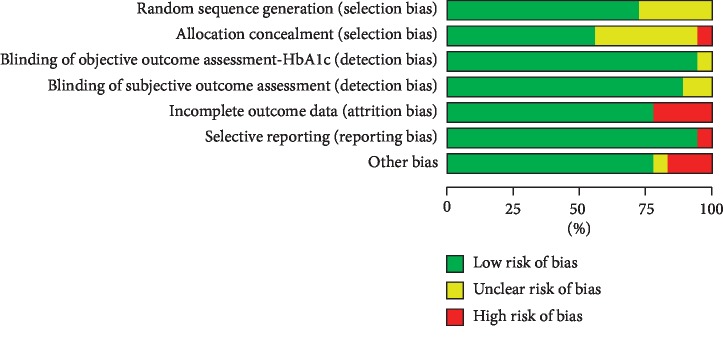
Risk of bias assessment across all included RCTs.

The risk of bias is shown in Figure 2. Ten studies reported adequate random sequence generation and allocation concealment [14, 26–28, 34–38, 40]. Five studies neither described adequate sequence generation nor mentioned allocation concealment [39, 41, 42, 44, 45]. Two studies reported a detailed description of randomization but did not indicate how the allocation concealment was implemented [25, 33]. In one study, a random sequence was generated by one member of the research team and did not mention allocation concealment, which can cause a high risk of bias due to poor allocation concealment [43]. The risk of bias for assessment of HbA1c and most of the subjective outcomes was considered low due to objective measurement and the use of standardized scales. Only seven studies provided intention-to-treat analyses [26, 27, 33–35, 37, 40]. Three studies did not report any information about dropout rates and reasons for dropouts [25, 28, 43], and one study provided incomplete psychological data and was at high risk of incomplete outcome data [40]. One study did not report all the outcomes mentioned in the published protocols or study design in the results, which can cause a high risk of selective outcome reporting [25]. Other sources of bias originated from the imbalance of baseline data on several demographic dimensions [36, 43, 45].

### 3.4. Effect of Interventions

Eighteen studies examined the effectiveness of SE. Of those studies, twelve targeted adolescent patients [26–28, 34–36, 38, 40–42, 44, 45] and six targeted adult patients [14, 25, 33, 37, 39, 43]. HbA1c was measured in all of those RCTs and was integrated in this meta-analysis according to the follow-up time after the intervention: short term (up to 6 months), medium term (up to 12 months), and long term (more than 12 months). Due to the considerable heterogeneity, pooling the results was not recommended in the meta-analysis. We performed a subgroup analysis according to age group, and the results were reported separately in adolescents and adults.

### 3.5. Effect of Interventions in Adolescent Patients

#### 3.5.1. HbA1c

**Figure 3:**
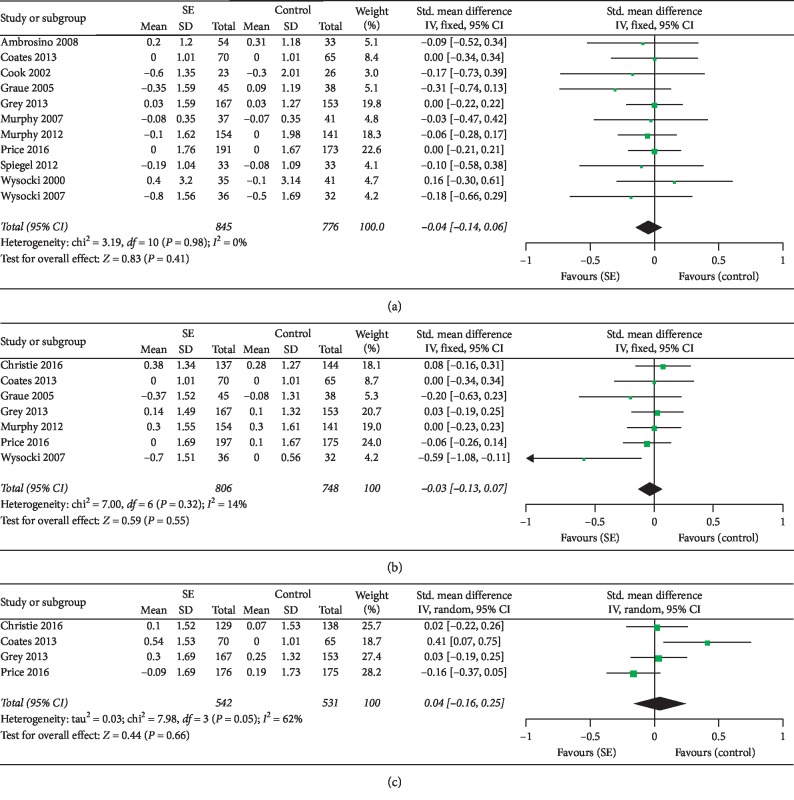
Forest plots showing the effect sizes of SE on HbA1c in adolescent patients with T1DM. (a) Short-term effect, (b) medium-term effect, and (c) long-term effect.

*(1) Short-Term Effect of SE on HbA1c*. Eleven studies examined the short-term effects of SE on HbA1c and were pooled in the meta-analysis [27, 28, 34–36, 38, 40–42, 44, 45]. There was no significant heterogeneity between studies (*I*^2^ = 0%); thus, a fixed-effects model was selected. The meta-analysis (Figure 3) showed no beneficial short-term effect of SE on HbA1c compared to control conditions (SMD = −0.04; 95% CI: −0.14 to 0.06; overall effect *P*=0.41).

*(2) Medium-Term Effect of SE on HbA1c*. Seven studies examined the medium-term effects of SE on HbA1c and were pooled in the meta-analysis [26–28, 35, 41, 42]. The heterogeneity between studies was low (*I*^2^ = 14%); thus, a fixed-effects model was selected for data synthesis. The meta-analysis (Figure 3) showed no beneficial medium-term effect of SE on HbA1c compared to control conditions (SMD = −0.03; 95% CI: −0.13 to 0.07; overall effect *P*=0.55).

*(3) Long-Term Effect of SE on HbA1c*. Four studies examined the long-term effects of SE on HbA1c and were pooled in the meta-analysis [26–28, 34]. With significant heterogeneity (*I*^2^ = 62%), the random-effects model was selected for data synthesis. The meta-analysis (Figure 3) showed no beneficial long-term effect of SE on HbA1c compared to control conditions (SMD = 0.04; 95% CI: −0.16 to 0.25; overall effect *P*=0.66).

### 3.6. Diabetes Self-Efficacy

**Figure 4:**
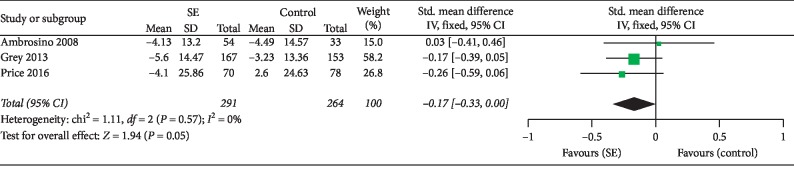
Effect size on changes in diabetes self-efficacy between the intervention and control groups in adolescent patients with T1DM.

The effect of SE on the diabetes self-efficacy scale in adolescent patients with type 1 diabetes was examined in three studies [27, 34, 38]. There was no significant heterogeneity between studies (*I*^2^ = 0%); thus, a fixed-effects model was selected. The meta-analysis (Figure 4) did not show a significant effect of SE on diabetes self-efficacy compared to control conditions (SMD = −0.17; 95% CI: −0.33 to 0.00; overall effect *P*=0.05).

### 3.7. Quality of Life

The effect of SE on QOL in adolescent patients with type 1 diabetes was examined in seven studies [26, 27, 34, 35, 38, 40, 42]. Because of the differences in measurement scales, scoring methods, and insufficient data, the QOL results in those studies were presented in different patterns and were not suitable for pooling in the meta-analysis. Therefore, we performed a narrative synthesis. Price et al. [27] reported that the intervention group showed significant improvement in total generic QOL scores compared with controls in the short and medium term (*P*=0.04). Graue et al. [42] reported improvement in two components of QOL (diabetes-related impact, *P*=0.018; diabetes-related worries, *P*=0.004) among older adolescents compared with those in the control condition. However, there were no statistically significant improvements in QOL in the other five studies [26, 34, 35, 38, 40].

### 3.8. Effect of Interventions in Adult Patients

#### 3.8.1. HbA1c

**Figure 5:**
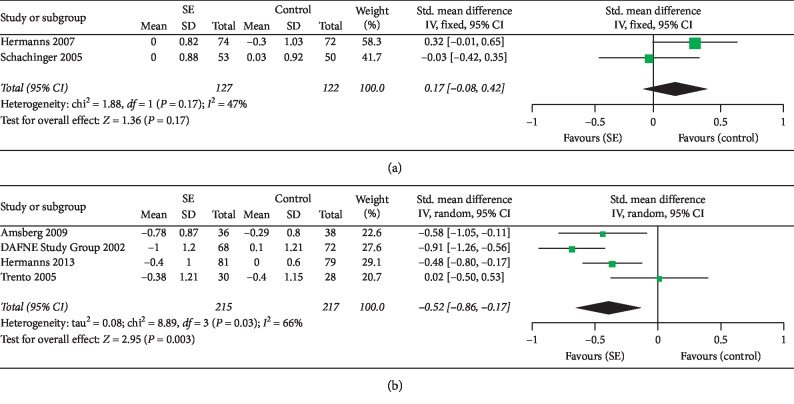
Forest plots showing the effect sizes of SE on HbA1c in adult patients with T1DM. (a) Baseline HbA1c <7.5% and (b) baseline HbA1c ≥7.5%.

Six studies investigated the effectiveness of SE in adult patients with type 1 diabetes [14, 25, 33, 37, 39, 43], and HbA1c was measured in all of those RCTs. Since the results in those RCTs could not be integrated in the meta-analysis due to considerable heterogeneity, we performed a subgroup analysis according to baseline HbA1c (HbA1c <7.5% or ≥7.5%). There were two studies with baseline HbA1c <7.5% [25, 39]. With moderate heterogeneity between studies (*I*^2^ = 47%), a fixed-effects model was selected for data synthesis. The meta-analysis (Figure 5) did not show a significant effect of SE in adults with low baseline HbA1c compared to control conditions (SMD = −0.17; 95% CI: −0.08 to 0.42; overall effect *P*=0.17). There were four studies with baseline HbA1c ≥7.5% [14, 33, 37, 43]. With significant heterogeneity (*I*^2^ = 66%), a random-effects model was selected for data synthesis. The results (Figure 5) showed a significant improvement in adults with baseline HbA1c no less than 7.5% compared to controls (SMD = −0.52; 95% CI: −0.86 to −0.17; overall effect *P*=0.003).

### 3.9. Well-Being

**Figure 6:**
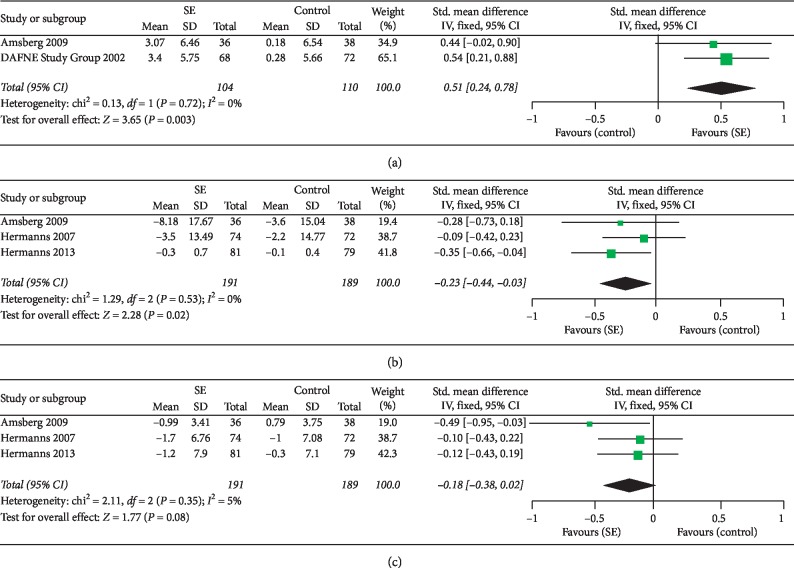
Forest plots showing effect size in changes in outcomes after intervention in adult patients with T1DM. (a) Well-being, (b) psychological distress, and (c) depression.

The effect of SE on well-being in adult patients with type 1 diabetes was examined in two studies [14, 37]. There was no significant heterogeneity between studies (*I*^2^ = 0%); thus, a fixed-effects model was selected for data synthesis. The meta-analysis (Figure 6) showed that SE had a significant effect on well-being compared to control conditions (SMD = 0.51; 95% CI: 0.24 to 0.78; overall effect *P* < 0.001).

### 3.10. Psychological Distress

The effect of SE on psychological distress in adult patients with type 1 diabetes was examined in three studies [33, 37, 39]. There was no significant heterogeneity between studies (*I*^2^ = 0%); thus, a fixed-effects model was selected for data synthesis. The meta-analysis (Figure 6) showed that SE had a significant effect on psychological distress compared to control conditions (SMD = −0.23; 95% CI: −0.44 to −0.03; overall effect *P*=0.02).

### 3.11. Depression

The effect of SE on depression in adult patients with type 1 diabetes was examined in three studies [33, 37, 39]. The heterogeneity between studies was low (*I*^2^ = 5%); thus, a fixed-effects model was selected for data synthesis. The meta-analysis (Figure 6) did not show a significant effect of SE on depression compared to control conditions (SMD = −0.18; 95% CI: −0.38 to −0.02; overall effect *P*=0.08).

### 3.12. Quality of Life

The effect of SE on QOL in adult patients with type 1 diabetes was examined in four studies [14, 25, 39, 43]. Given the different measurement scales and scoring methods, the QOL results were not suitable for pooling in the meta-analysis. Therefore, a narrative synthesis was performed. The DAFNE Study Group [14] reported that the intervention group showed significant improvement in overall QOL compared with the control group in the short term (*P* < 0.01). Trento et al. [43] found significant improvement in QOL (*P* < 0.001), whereas QOL was worsened among control patients. Hermanns et al. [33] and Schachinger et al. [25] found no overall effect of intervention on QOL.

## 4. Discussion

This systematic review and meta-analysis examined the effects of SE on glycemic control and various psychological outcomes. Based on a systematic literature review, 18 RCTs that met the inclusion criteria were selected from a set of 1741 references.

### 4.1. Effect of SE in Adolescent Patients

Our meta-analysis synthesized the data from twelve RCTs targeting adolescent patients with type 1 diabetes. We found no significant effect of SE on HbA1c in the short, medium, and longer term, and we also found no significant improvement in diabetes self-efficacy compared with control conditions. Based on the narrative synthesis, this review showed that SE may not improve QOL among adolescent patients with type 1 diabetes in most studies. These results indicated that SE may not be suitable for adolescent patients with type 1 diabetes. The reasons why SE has no significant effect in adolescent patients can be viewed from two perspectives. For patients, the reason might be due to the difficulties in implementing interventions in this age group [46]. The way of thinking in adolescent patients is still in the developmental stages and is not yet mature. The barriers to effectively managing type 1 diabetes include lacking knowledge and a better understanding of the disease, the therapeutic regimen, and potential complications of diabetes. The population was adolescents, and many researchers mentioned the poor compliance with the intervention. Many of the unique issues that account for the deterioration in diabetes management occur in this transitional period. Poor self-control, which may be related to behavioral, emotional, and cognitive changes during adolescence [47], prevents them from achieving better glycemic control as required by their doctors. For studies, the reasons might be due to a lack of effectiveness of the intervention itself [48]. Our research found that the content of many interventions focused on carbohydrate counting and/or insulin adjustment that may be more complicated for adolescent patients to understand. In most studies, psychological approaches were not incorporated into the delivery of SE to increase compliance and develop confidence and motivation to change. The other reason from a research point of view might be explained by the short observation time in most studies. Our findings supported the view of Couch et al. [49] that any particular educational intervention targeted to adolescent patients with type 1 diabetes may not improve metabolic control or QOL. Our results of this subgroup analysis are consistent with a recent systematic review [50], which indicated that SE could not be strongly recommended for current pediatric clinical practice but should be matched with the right diabetes patient group. However, the validity of the results was weakened by incorporating nonrandomized controlled trials and unclear definitions of SE, leading to the incorporation of studies that did not meet the key criterion of SE, and this systematic review did not discuss the long-term effects of SE. Our findings are different from those of a review [51] that recommended SE to all adolescent and adult patients with type 1 diabetes. This discrepancy might be because the study was only a review and did not provide sufficient evidence. Our systematic review and meta-analysis incorporated only RCTs and assessed a broader variety of outcomes, including biomedical and psychosocial outcomes, all of which increase the reliability of the study. The effects of SE on HbA1c were evaluated according to the follow-up time after intervention.

### 4.2. Effect of SE in Adult Patients

This meta-analysis integrated the data from six RCTs targeting adult patients with type 1 diabetes. From the results of the subgroup analysis of baseline HbA1c (HbA1c <7.5% or ≥7.5%), we found no significant effect of SE on lower baseline HbA1c level, but there was a statistically significant benefit of structured education on higher baseline HbA1c levels. This finding could be because people with lower baseline HbA1c levels have better self-management ability and thus have better glycemic control. In addition, SE significantly improved well-being and psychological distress but had no significant effect on the improvement of depression. This observation could be due to most interventions focusing on the management of blood glucose while ignoring psychological conditions. Based on the narrative synthesis, we found inconsistent results for QOL, with two studies showing improvement in quality of life and the other two studies showing a nonsignificant effect. Our results indicate that SE should be implemented in adult patients with type 1 diabetes, especially in adults with a higher baseline HbA1c level. The findings are consistent with the guidelines of the National Institute for Health and Care Excellence (UK) [15] stating that SE should be available to adult patients with type 1 diabetes but made little difference. However, we found a novel indication that adult patients with a higher baseline HbA1c level might benefit from SE. Future work should consider the personality characteristics of patients.

### 4.3. Strengths and Limitations

This meta-analysis has several strengths. Although a systematic review of SE programs for children with type 1 diabetes has been published, to our knowledge, this paper is the first meta-analysis that included only RCTs for all age groups, guaranteeing the highest quality of evidence [52]. Second, all of the included RCTs measured HbA1c values, which is an objective value and an acknowledged indicator of glycemic control that can better illustrate the effect of SE in a meta-analysis. We also assessed a broader variety of psychosocial outcomes that can evaluate the effects of SE more comprehensively. Third, the effects of SE on HbA1c in adolescent patients were evaluated in the short, medium, and longer term, focusing on the benefits of SE at different time periods.

Our meta-analysis still has some limitations. First, blinding of participants and personnel is not feasible for trials of SE. Second, we excluded the studies from which the HbA1c results were unable to be integrated in the meta-analysis. Although we corresponded with the original author, there were still two authors who did not reply. Third, a subgroup analysis on follow-up time after intervention could not be performed in adults due to the considerable heterogeneity and lack of RCTs. Finally, because of the differences in measurement and the insufficient data, some relevant parameters for evaluating the effects of SE, such as severe hypoglycemia and TIR, are not suitable for pooling in the meta-analysis. Finally, because of the differences in measurement and the insufficient data, some relevant parameters for evaluating the effects of SE, such as severe hypoglycemia and TIR, are not suitable for pooling in the meta-analysis.

### 4.4. Recommendations for Further Research

The overall results of our meta-analysis suggest that SE should be implemented in the appropriate patient group based on personal characteristics and development, especially for patients with poor glycemic control. There is insufficient evidence to recommend SE programs for adolescent patients with type 1 diabetes. Future studies are warranted to explore the reasons why many SE programs have insufficient effects on adolescent patients by using various approaches, such as in-depth interviews or experimental methods, thus modifying the existing SE programs or developing new SE programs more suitable to this age group. Our research suggests that the content and form of future SE programs should be more simple and flexible, consistent with the cognitive and psychological characteristics of adolescents, for example, adding cartoon elements, using role-playing or other activities in the delivery of SE. For adult patients, SE programs play an active role, especially in adults with a higher baseline HbA1c level. More well-designed RCTs are needed to evaluate the effects of SE in adults with type 1 diabetes.

## 5. Conclusions

In conclusion, our meta-analysis indicates that SE can significantly improve glycemic control among adult patients with higher HbA1c levels but has no significant effect on glycemic control, diabetes self-efficacy, or QOL in adolescent patients with type 1 diabetes. More attention should be paid to why many SE programs have no sufficient effects in adolescent patients. Additionally, more well-designed RCTs are needed to better assess the effects of SE in adult patients, and the follow-up time should be much longer. These findings are instructive for clinical practice. Overall, clinicians should consider personal characteristics and development when implementing SE programs.

## Figures and Tables

**Table 1:** Characteristics of the included studies.

First author, year (ref.)	Inclusion criteria	Intervention	Participant numbers study/control	Mean (SD) age (years)	Mean (SD)% HbA1c at baseline	Control group	Interventionist	Assessment/follow-up	Outcome measures
Christie et al. 2016 [26]	Age 8–16 years with type 1 diabetes, mean 12-month HbA1c ≥ 8.5%	CASCADE consisted of four monthly modules, incorporating motivational interviewing and solution-focused brief therapy, groups of 3 to 4 families	180/182	13.15 (2.1)	9.95 (1.5)	Usual care	Pediatric clinical nurse specialist, diabetes team member	At baseline and 12 and 24 months	HbA1c; QOL

Price et al., 2016 [27]	Age 11–16 years with type 1 diabetes for at least one year	KICK–OFF, a 5-day group education that focused on carbohydrate counting and insulin adjustment in everyday life, groups of 8 participants	199/197	13.8 (1.5)	9.2 (1.7)	Usual care	A nurse, a dietician, one local team member	At baseline and 6, 12, and 24 months	HbA1c; QOL

Coates et al. 2013 [28]	Age 13–19 years with type 1 diabetes for at least 12 months	CHOICE, which focused on insulin adjustment to liberate diet and lifestyle, delivered in four consecutive weekly sessions for 3 hours, groups of 8 participants	70/65	15.4 (1.8)	8.9 (1.5)	Usual care	A diabetes specialist nurse and a trained psychologist	At baseline and 1, 3, 6, 12 and 24 months	HbA1c

Hermanns et al. 2013 [33]	Age 18–75 years with type 1 diabetes for more than 1 month, BMI >20 and <40 kg/m^2^ and HbA1c ≥7.0% and ≤13.0%	The PRIMAS program consisted of 12 lessons of 90 min each, based on a self-management empowerment approach; groups of 3 to 8 participants, 6-week period with 2 sessions per week.	81/79	45.3 (13.6)	8.2 (1.0)	Standard care	Certified diabetes educators	At baseline, end of the intervention, 6 months posttreatment	HbA1c; diabetes-related distress; depression; diabetes self-efficacy

Grey et al. 2013 [34]	Age 11–14 years with type 1 diabetes for at least 6 months	TeenCope consisted of five weekly sessions, delivered in a group-based in-person format	167/153	12.3 (1.1)	8.46 (1.42)	Attention control	Health professionals	At baseline and 3, 6, 12, and 18 months	HbA1c; QOL

Murphy et al. 2012 [35]	Adolescents with type 1 diabetes for more than 12 months	FACTS consisted of six 90 min monthly sessions, incorporating skills training and family teamwork, groups of 4 to 6 families	158/147	13.1 (1.9)	9.3 (1.9)	Usual care	Health professionals	At baseline and 9, 12, and 18 months	HbA1c; QOL diabetes-related distress; well-being

Spiegel et al. 2012 [36]	Age 12–18 years with type 1 diabetes for more than 1 year	Education program including an interactive 90 min class, which focused on carbohydrate counting, attended by 1 to 2 participants.	33/33	15.1 (2.8)	8.3 (1.1)	Usual care	A registered dietitian/certified diabetes educator (RD/CDE)	At baseline and 3 months	HbA1c
Amsberg et al. 2009 [37]	Age 18–65 years with type 1 diabetes at least two years, BMI <30 kg/m^2^, HbA1c >7.5% during the last year	The CBT-based intervention consisted of a basic intervention program (weeks 0–8) and a structured maintenance program (weeks 9–48) that focused on behavior changes, delivered in groups of 4–6 patients	46/48	41.2 (12.3)	8.5 (0.8)	Usual care	A diabetes specialist nurse and a trained psychologist	At baseline and end of intervention	HbA1c; diabetes-related distress; well-being; depression

Ambrosino et al. 2008 [38]	Age 8–12 years, diagnosed with type 1 diabetes and treated with insulin for at least 6 months	CST consisted of six 1.5-hour sessions, conducted with groups of 2 to 5 children	54/33	9.9 (1.4)	7.0 (1.3)	Attention control	A mental health professional	At baseline and 3 months	HbA1c; QOL

Hermanns et al. 2007 [39]	Age 18–70 years with type 1 diabetes and at least one episode of severe hypoglycemia in the previous 12 months	HyPOS consisted of five 90 min weekly lessons for the purpose of treatment of impaired hypoglycemia awareness	74/72	46.0 (12.5)	7.3 (1.0)	Standard care	Diabetologists and diabetes educators	At baseline, 6 months after the end of the intervention	HbA1c; QOL; diabetes-related distress; depression

Murphy et al. 2007 [40]	Age 8–16 years with type 1 diabetes for more than 12 months	FACTS consisted of four 3-hour monthly sessions—two were skills-based and two based on social learning theory, each session for 1 hour, groups of 3 to 5 families	37/41	12.5 (2.4)	9.1 (1.2)	Usual care	Diabetes multidisciplinary team	At baseline and 6 and 12 months	HbA1c; QOL

Wysocki et al. 2007 [41]	Age 11–16 years with type 1 diabetes at least 2 years, HbA1c ≥8.0%	BFST-D consisted of four components that focused on A1C, treatment adherence, and diabetes-related family conflict. Groups of three to five families received 12 BFST-D sessions over 6 months	32/36	14.0 (1.9)	9.6 (1.6)	Standard care	Psychologists, a licensed clinical social worker	At baseline and 6, 12, and 18 months	HbA1c

Graue et al. 2005 [42]	Age 11–17 years with type 1 diabetes	A 15month structured educational and counseling program consisting of three 3-hour group visits, three 45 min individual consultations, and one meeting for the parents; groups of 4 to 9 participants	55/46	14.4 (1.6)	9.5 (1.5)	Usual care	A physician, diabetes nurse specialist, clinical psychologist, dietician, and social worker	At baseline and 15 months	HbA1c; QOL
Schachinger et al. 2005 [25]	Adults with type 1 diabetes	BGAT III, consisting of eight weekly sessions, was delivered in groups of 5–12 subjects, that focused on improving recognition and management of extreme blood glucose levels	56/55	46.3 (13.8)	6.9 (0.9)	Attention control	A physician/psychologist team	At baseline, 1–6 months and 7–12 months postintervention	HbA1c; QOL

Trento et al. 2005 [43]	Age <70 years with type 1 diabetes	Group care consisted of 9 sessions delivered in groups of 6-7 patients. Each class lasted 40–50 min	30/28	29 (10.9)	8.7 (1.2)	Usual care	A doctor and a psychologist	At baseline and 3 years	HbA1c; QOL

DAFNE study group, 2002 [14]	Age over 18 years with type 1 diabetes for more than two years, HbA1c (7.5–12%)	DAFNE, a 5-day program that focused on insulin adjustment to suit diet and lifestyle, groups of 6–8 people	68/72	40 (9)	9.3 (1.1)	Usual care	Diabetes specialist nurses and dietitians	At baseline and 6 months	HbA1c; QOL; well-being

Cook et al. 2002 [44]	Age 13–17 years with type 1 diabetes for at least 1 year	The CHOICE program, conducted over 6 weeks with 2-hour weekly sessions, delivered in groups of 6 patients	26/27	14.6 (1.3)	9.1 (1.8)	Usual care	Experienced teachers	At baseline and 6 months postenrollment	HbA1c

Wysocki et al. 2000 [45]	Age between 12 and 16.75 years with type 1 diabetes for at least year	BFST consisted of 4 therapy components (10 sessions) matched to families' treatment needs	41/38	14.4 (1.3)	11.8 (3.2)	Usual care	Licensed psychologists	At baseline and 3 months posttreatment	HbA1c

CASCADE, The Child and Adolescent Structured Competencies Approach to Diabetes Education; KICK–OFF, Kids in Control of Food; QOL, quality of life; CHOICE, Carbohydrate, Insulin, Collaborative Education; PRIMAS, Programme for diabetes education and treatment for a self-determined living with type 1 diabetes; FACTS, Families and Adolescents Communication and Teamwork Study; CBT, Cognitive Behavior Therapy; CST, Coping Skills Training; HyPOS, Hypoglycaemia Treatment Programme; BFST-D, Behavioral Family Systems Therapy for Diabetes; BGAT, Blood Glucose Awareness Training; DAFNE, Dose Adjustment for Normal Eating; BFST, Behavioral Family Systems Therapy.

## Data Availability

The data used to support the findings of this study are provided in Table 1 in the article.
